# Myrmecovory in Neotropical primates

**DOI:** 10.1007/s10329-021-00946-2

**Published:** 2021-09-29

**Authors:** Nadja I. Risch Ferreira, Manfred Verhaagh, Eckhard W. Heymann

**Affiliations:** 1 Abteilung Soziobiologie/Anthropologie, Georg-August Universität Göttingen, Kellnerweg 6, 37077 Göttingen, Germany; 2 Verhaltensökologie and Soziobiologie, Deutsches Primatenzentrum-Leibniz-Institut für Primatenforschung, Kellnerweg 4, 37077 Göttingen, Germany; 3 Staatliches Museum für Naturkunde, Erbprinzenstr. 13, 76133 Karlsruhe, Germany

**Keywords:** Platyrrhini, Formicidae, Insectivory, Predation, Foraging technics, Ant defences

## Abstract

Ants are the dominant group of animals in many habitats, particularly in tropical rainforests. High abundance and formation of large colonies convert them into a potential food source for a broad spectrum of animals. In this paper we review myrmecovory (consumption of ants) in Neotropical primates. Myrmecovory has been reported from 57 taxa (species + subspecies) out of 217 species of Neotropical primates, representing 18 out of 22 genera. The proportion of ants in the animal portion of the diet is highest amongst members of the genera *Cebus*, *Sapajus*, *Cheracebus* and *Plecturocebus*, but generally low in callitrichids, large pitheciids (*Cacajao*, *Chiropotes*) and atelids. Ants from seven subfamilies of Formicidae (out of 13 subfamilies found in the Neotropics) are consumed, including taxa with and without functional sting and with varying other defences. Foraging technics employed in myrmecovory range from picking ants from open substrates to extractive foraging involving the destruction of ant nests or shelters, but tool use has not been reported. We conclude that myrmecovory is widespread amongst Neotropical primates but on average contributes only a minor proportion of the diet. The diversity of foraging technics employed and lack of tool use in Neotropical primate myrmecovory, even for ants with functional stings and aggressive biting, suggests that tool use for myrmecovory in hominids has not evolved in response to ant defences but is a consequence of enhanced cognitive skills that evolved under other selection pressures.

**Supplementary Information:**

The online version contains supplementary material available at 10.1007/s10329-021-00946-2.

## Introduction

Insects are included in the diet of almost all primates and are procured either through active foraging or through the inadvertent consumption of insects imbedded in plant material like e.g. fig wasps in syconia (Harding 1981; McGrew 2014; O'Malley and McGrew 2014). Insects can provide nutrients (fat, protein) and vitamins not present in large concentrations in typical primate diets (Rothman et al. 2014). Also, differences in primates’ insect foraging strategies are crucial factors contributing to niche segregation in primate communities, particularly in the Neotropics (Terborgh 1983; Nadjafzadeh and Heymann 2008). Depending on their lifestyle, insects represent scattered (e.g., katydids) or clumped (e.g., colony-living insects) resources. Colony-living Hymenoptera can be a profitable resource, particularly for larger primates (Isbell et al. 2013; Rothman et al. 2014). However, hymenopterans possess diverse anti-predator defences, including stings and venoms (Hermann and Blum 1981). Despite these defences, ants are preyed upon by a broad spectrum of mammals, including several specialists (e.g. Edentata, Pholidota) and many opportunists, including primates (Redford 1987). Recently, Schmidt (2014 p. 12) suggested that “predators have been a strong component of the selection pressure in the evolution of painful and toxic bee, wasp and ant stings and these insects, in turn, have influenced hunting behaviour and learning in at least higher primates”. The use of sticks and grass blades by chimpanzees for feeding on ants is probably the most well-known example of such an influence (McGrew 1974; Humle and Matsuzawa 2002).

In the Neotropics, ants are the dominant animal taxon in terms of abundance and biomass (Fittkau and Klinge 1973; Wilson 1987; Tobin 1995; Davidson and Patrell-Kim 1996; Harada and Adis 1997; Dejean and Corbara 2003; Verhaagh 2005). It is therefore not surprising that the Neotropics harbour a broad spectrum of vertebrates that prey on ants, including the Dasypodidae (armadillos) and Myrmecophagidae (anteaters) amongst mammals; Formicariidae (antthrushes) and many other birds (Macedo Mestre et al. 2010; Sherry et al. 2020); Tropiduridae (lizards) and Gymnophthalmidae (spectacled lizards) amongst reptiles (Vitt et al. 1998; Goldberg et al. 2009); and many species of Dendrobatidae (poison frogs), Microhylidae (narrow-mouthed frogs) and Bufonidae (toads) amongst amphibians (Duellman 1978; Toft 1980). Many Neotropical primates (Platyrrhini), particularly the small and medium-sized ones (i.e., up to around 3.5 kg body mass), show a high degree of insectivory (Ford and Davis 1992; Rosenberger 1992), in line with the Jarman-Bell principle that smaller animals should consume higher-quality food (Gaulin 1979). Reports on ants in their diet are spread through the primatological literature. It is therefore timely to review patterns of myrmecovory in platyrrhines and to specifically examine the following questions: (a) How widespread is myrmecovory amongst platyrrhines? (b) What is the significance of ants in the diets of platyrrhines? (c) Does body mass explain eventual variation in myrmecovory amongst platyrrhines? (d) Which ants are consumed by platyrrhines? (e) Is platyrrhine myrmecovory influenced by ant defences? (f) How are ants procured by platyrrhines, i.e., which foraging technics are employed in myrmecovory?

## A note on terminology: myrmecovory or myrmecophagy?

What is the correct technical term ant eating, myrmecophagy or myrmecovory? An inquiry into the etymology revealed the following: “-phagy” is derived from “-phagous”, which is a word-forming element meaning “eating, feeding on” which stems from the Greek “-phagos” meaning “eater of”. “-vory” is derived from “-vorous”, a word-forming element meaning “eating” which itself is derived from the Latin word “vorare”, meaning “devour, swallow” (https://www.etymonline.com [accessed 10 May 2021]). Thus, both terms have the same meaning. In line with the general use of “-vory” in primatology (e.g., frugivory, folivory, etc.), we opt for using myrmecovory.

## Methods

We conducted a thorough online literature search using popular databases (Web of Science, Google Scholar, PrimateLit). Our searches combined the term "ants" with the names of New World primate genera. We used the function “cited by” to scrutinize articles for the detection of additional relevant work. We also browsed the literature on platyrrhine feeding ecology for information on the prey spectrum.

From pertinent articles we extracted the following information:

- Primate species. We used the current name, based on the most recent taxonomic revisions (Lynch Alfaro et al. 2012, 2015; Byrne et al. 2016; Rylands et al. 2016).
- Proportion of prey in the diet and proportion of ants in the prey. If articles reported the proportion of different prey taxa in the overall diet, we summed up all prey and calculated the proportion of ants from this sum. We did not include % of prey if this was based on foraging time rather than feeding records or feeding time, as this may inflate/overestimate the relative proportion of prey in the diet. If consumption of ants was reported without quantitative information, we scored myrmecovory as present (+).
- Ant taxa (subfamily, genus or species) reported as prey. For our analyses, we only used subfamily and genera, as the ants were rarely identified on the species level.
- Foraging technics used to procure ants.

When articles reported prey spectra at least on an ordinal or familial level (e.g., butterflies, beetles, katydids) but did not mention ants, we assumed that these were in fact not part of the diet; we extracted information on the proportion of prey from these articles and scored myrmecovory as absent (−).

Neotropical primate body mass was calculated as the midpoint of ranges provided in Mittermeier et al. (2013). We plotted the proportion of ants in the animal prey against primate body mass in Statistica 13 (Dell Inc. 2015). For those species for which quantitative information on the proportion of ants was available from two or more studies (e.g., *Cebus cuscinus*), we used the midpoint of the range. For *Callithrix jacchu*s, this proportion included termites (Souto et al. 2007); therefore, we used half the value indicated in the source for the analyses. Species for which myrmecovory was scored as absent (see above) were entered with a proportion of 0. As the plot of the proportion of ants vs. body mass suggested a relationship between these variables for Cebidae and Pitheciidae (but not for Callitrichidae and Atelidae), we performed regression analyses in Statistica 13 (Dell Inc. 2015).

We researched the pertinent literature for information on ant defences.

For primate taxonomy, we consulted the most recent listing of families, genera, species and subspecies of the IUCN Primate Specialist Group (http://www.primate-sg.org). In the database (Supplementary Table 1), we used the currently recognized name of each species and added the name used in the original publications in brackets.

## Results

### Distribution and significance of myrmecovory in Neotropical primates

Myrmecovory has been reported from members of all five families of Neotropical primates, for 57 out of 217 (26%) species and 18 out of 22 (82%) genera of Neotropical primates (Supplementary Table 1). The only genera for which myrmecovory has not been observed in the wild are *Brachyteles*, *Callibella*, *Callicebus* and *Leontopithecus*. The proportion of ants in the animal part of the diet varies between < 1% in *Leontocebus weddelli* and *Saimiri oerstedii* and 69.5% in *Plecturocebus oenanthe*. On the familial level, myrmecovory is more pronounced in Cebidae and Pitheciidae compared to Callitrichidae and Atelidae. In most cases, ant consumption was directly observed; in some studies, it was concluded from analyses of stomach contents (Ayres and Nessimian 1982; Milton and Nessimian 1984; Ferrari et al. 1993; Silvestre et al. 2016) or from metagenomic analyses of faecal matter (Pickett et al. 2012; Mallott et al. 2015).

### Primate body mass and myrmecovory

**Fig. 1:**
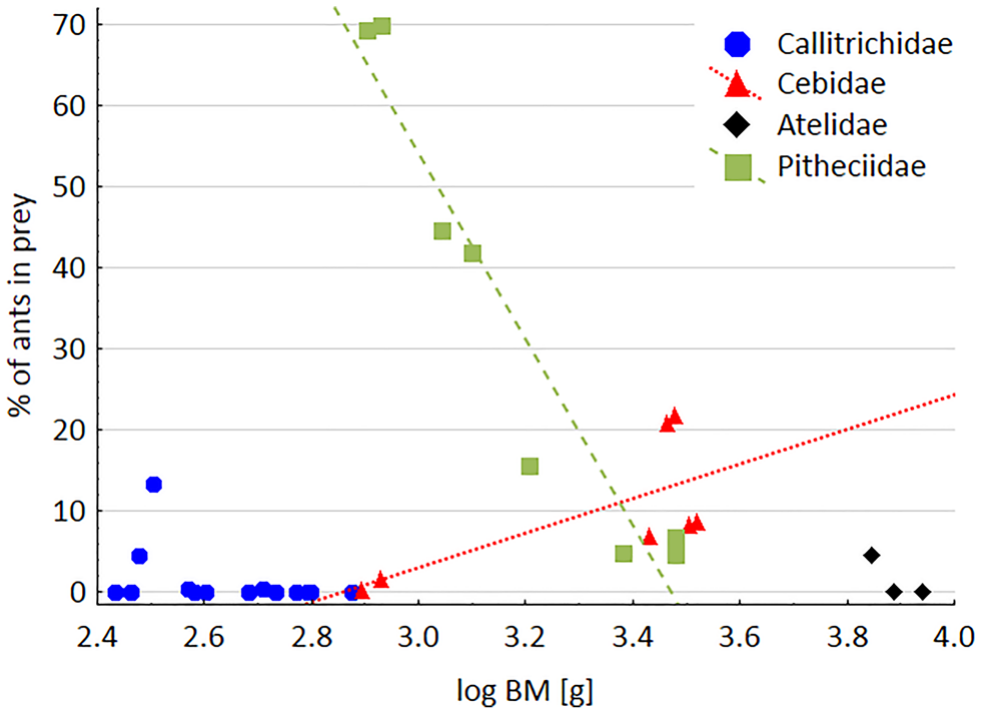
Proportion of ants in the animal prey in relation to primate body mass

The proportion of ants in the prey spectrum is lowest for small and large Neotropical primates, and higher for species between ca. 800 and ca. 3200 g (Fig. 1). There seems to be a trend for increasing ant consumption with increasing body mass in Cebidae (*R*^2^ = 0.39, *p* = 0.078) and decreasing ant consumption with increasing body mass in Pitheciidae (*R*^2^ = 0.61, *p* = 0.008). If only studies with a duration of ≥ 6 months are included, the same pattern emerges (Cebidae: *R*^2^ = 0.53, *p* = 0.062; Pitheciidae: *R*^2^ = 0.89, *p* = 0.003).

### Ants consumed by Neotropical primates in relation to defences

**Fig. 2:**
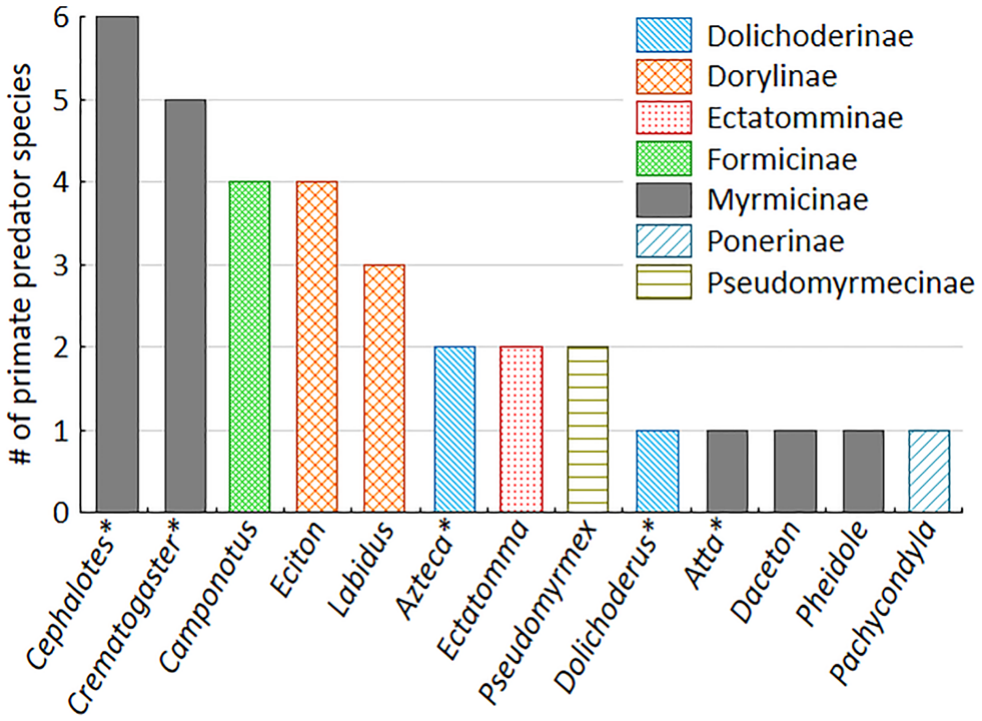
Ant genera included in the diet of Neotropical primates and the number of Neotropical primate species preying upon these ants. Genera marked with an asterisk (*) lack a functional sting. For other defences see Supplementary Table 2

Ants from 13 genera and seven subfamilies (out of 13 subfamilies found in the Neotropics, Bolton 2021) are included in the prey spectrum (Fig. 2). Five genera from the subfamily Myrmicinae are preyed upon; all other ant subfamilies are represented as prey only by one or two genera. The ant genera vary in the type of defences. *Azteca*, *Dolichoderus*, *Camponotus*, *Cephalotes*, *Crematogaster* and *Atta* lack a functional sting, while *Eciton*, *Labidus*, *Daceton*, *Pheidole*, *Ectatomma*, *Pachycondyla* and *Pseudomyrmex* possess (large) functional stings and the latter three inject painful venoms. Others mainly rely on aggressive biting, spraying of repellents or spiny exoskeletons (Supplementary Table 2). Ant genera with functional stings are preyed upon by 17 primate species, those without a functional sting by 15 primate species (Fig. 2). The scarcity of detailed data on rates of myrmecovory does not allow us to examine whether the type of defence has an influence on predation rates.

### Foraging technics employed in myrmecovory

Details on the foraging technics employed in myrmecovory are reported for only 21 Neotropical primate species. These technics can be grouped into six categories: (a) grab or lick ants from open substrates (e.g., leaf surface); (b) capture swarming alate ants from air; (c) “fishing” with the tail; (d) unroll fresh and/or dry leaves, and turn around branches with leaves; (e) search in the leaf litter; (f) scratch open or destroy ant nests; (g) bite or break open closed substrates. Most species employ only one technic, a few employ two (Table 1). Destructive technics (f, g) are only reported from different species of *Cebus* and *Sapajus*, and from Peruvian red uakaris, *Cacajao calvus ucayalii*. In wedge-capped capuchins, *Cebus olivaceus*, the speed of grabbing depends on the ants taken: *Cephalotes* ants (spiny, non-functional sting) are taken slowly and methodologically, *Camponotus* (biting and spraying formic acid as repellent) and *Pachycondyla* (functional sting and painful toxin) are rapidly grabbed and put into the mouth (John G. Robinson, pers. communication, 9 Dec 1988). San Martín titi monkeys, *Plecturocebus oenanthe*, follow smooth-billed anis, *Crotophaga ani*, to feed on ants (DeLuycker 2012).

**Table 1:** Foraging technics employed by Neotropical primates in myrmecovory

Open substrates			Closed substrates			
Grab or lick from open substrate w/hand or mouth	Capture from air	“Fish” w/tail	Unroll fresh or dry leaves; turn around branches w/leaves; take from hollows	Search in leaf litter	Scratch or destroy ant nests	Bite or break open closed substrates^a^
*Saguinus mystax* *Callimico goeldii* *Callithrix penicillata* *Cebus olivaceus* *Sapajus nigritus* *Cheracebus lugens* *Plecturocebus cupreus* *Pithecia inusta* *Pithecia monachus* *Chiropotes satanas*^b^	*Sapajus libidinosus*	*Plecturocebus toppini*	*Cebuella pygmaea* *Cebus imitator* *Cebus kaapori* *Saimiri boliviensis boliviensis* *Saimiri oerstedii* *Plecturocebus oenanthe*	*Callithrix aurita*	*Sapajus libidinosus* *Cacajao melanocephalus*	*Cebus cuscinus* *Cebus imitator* *Cebus kaapori* *Cebus olivaceus* *Sapajus macrocephalus* *Sapajus xanthosternos* *Cacajao calvus ucayalii*

The number of genera in this table (12) is lower than the overall number of genera from which myrmecovory has been observed (18), as information on foraging technics is not available for all species

^a^Hollow branches and lianas, dry palm leave stems, *Acacia* thorns, bamboo, bark of deadwood

^b^Ants eaten from arm

## Discussion

Our literature review revealed that myrmecovory (a) is widespread amongst Neotropical primates; (b) contributes variable proportions to the prey spectrum, with ants being most strongly represented in the diets of capuchin monkeys and some titi monkeys; (c) tends to correlate positively with body mass in Cebidae and negatively in Pitheciidae; (d) involves species from 13 genera (representing seven of the 13 subfamilies of Formicidae in the Neotropics) which (e) do or do not possess functional stings; and (f) involves a broad spectrum of foraging technics.

The absence of myrmecovory in muriquis, *Brachyteles*, is not surprising, as this is the most folivorous platyrrhine (Rosenberger and Strier 1989); highly folivorous primates rarely consume any substantial amounts of animal prey at all (Chivers and Hladik 1980), in line with the Jarman-Bell principle (Gaulin 1979). In contrast, the lack of any report on myrmecovory in wild lion tamarins, *Leontopithecus*, and Atlantic Forest titi monkeys, *Callicebus*, is unexpected. Lion tamarins intensively forage for prey and include arthropods in their diet (Rylands 1989; Dietz et al. 1997; Passos and Keuroghlian 1999). In captivity, golden lion tamarins, *Leontopithecus rosalia*, readily fed on two species of *Atta* ants offered to them (Coimbra-Filho 1981). Some species of Atlantic titi monkeys, *Callicebus*, include insects in their diet while others do not, but prey taxa are rarely reported (Heiduck 1997; Barbosa Caselli and Setz 2011; Souza-Alves et al. 2011). It is very unlikely that the lack of myrmecovory in *Callicebus* is due to the lack of relevant foraging technics (as suggested by a reviewer), as *C. nigrifrons* has been shown to search for and capture prey on trunks and branches, to unroll dead and green leaves, and to grab flying insects, as Amazonian titi monkeys do (Heymann and Nadjafzadeh 2013). Thus, the lack of reports of myrmecovory from wild lion tamarins and other Neotropical primates may represent an observational bias. Ants (and other small prey items) are often grabbed rapidly and put into the mouth, so that identification of prey becomes impossible. Therefore, our estimate of the proportion of species for which myrmecovory is known is likely to be biased towards the conservative side. Increased use of metagenomic approaches (Pickett et al. 2012; Mallott et al. 2015) will help to solve the problem of identification of small prey items. In any case, the diversity and proportion of Neotropical primate taxa that include ants in their diet is much higher than that of Paleotropical primates. In African primates, myrmecovory is found in only 12 out of 25 (= 48%) genera (Isbell et al. 2013), considerably lower than the 82% of genera found in our study.

The proportion of ants in the diet of Neotropical primates is generally low, but for some species ants may actually represent the major prey taxon. In the absence of long-term data on the diet of most Neotropical primates, it remains unclear whether high proportions reported in some studies represent a general pattern or an exceptional year or period. For instance, in moustached tamarins, *Saguinus mystax*, rates of predation on frogs were generally very low, but in 1 year showed a 5–10-fold increase during a 3-month period (Lüffe et al. 2018). The lack of more long-term data also leaves the apparent positive correlation of the proportion of ants in the diet with body mass in cebids and negative correlation in pitheciids as a preliminary finding that needs to be verified or rejected with more data.

Neotropical primates prey both on ants with and without functional sting. Along with the variability of defences found in the ant genera preyed upon by Neotropical primates, this suggests that ant defences are not a major factor for prey choice. Panamanian white-faced capuchins, *Cebus imitator*, ignored *Atta* ants, which have no functional sting (Freese 1977). A wild-born juvenile monk saki, *Pithecia monachus*, fed on *Cephalotes atratus* (without functional stings), but rejected other ants with and without functional stings (Heymann and Bartecki 1990), which suggests that learning or experience might be involved in prey choice (Visalberghi and Addessi 2003). Guianan weeper capuchins, *Cebus olivaceus*, consume ants, despite being obviously affected by their defence (“yelps and slapping of hands and mouth”; John G. Robinson, pers. communication, 9 Dec 1988). This latter observation indicates a high positive ratio between nutritional benefits and physiological costs, i.e. the pain (Schmidt 2014) and eventual skin lesions caused by venom injection and bites.

Neotropical primates employ a broad spectrum of foraging technics in myrmecovory, ranging from picking ants from open substrates to extractive foraging involving the destruction of ant nests or of closed plant substrates (e.g., hollow *Acacia* thorns). Notably, tool use to procure ants has not been reported for any Neotropical primate, although bearded capuchins, *Sapajus libidinosus*, are known to use sticks to poke into termite nests (Falótico and Ottoni 2014). For the moment, it remains unclear whether this reflects an effective lack or simply a lack of observation and documentation.

The generally low levels of myrmecovory in Neotropical primates do not seem to support the notion of Schmidt (2014) that primates—as one group of ant predators—have played an important role for the evolution of ant defences. Also, the origin of ants dates back until the Middle Jurassic (140–168 m.y.a.), and occurrence and diversification of extant ant subfamilies date back to the Late Cretaceous or Early Paleocene (Brady et al. 2006; Moreau et al. 2006) making it more plausible that invertebrates (including other ants) imposed stronger selection pressure on defense methods than vertebrates. The lack of tool use in Neotropical primate myrmecovory (if confirmed by further observations) also does not clearly support the hypothesis of Schmidt (2014) that ants (and other Hymenoptera) have influenced hunting behaviour and learning in higher primates. Ants do not seem to be a critical or seasonal fall-back resource for any Neotropical primate. Therefore, the model by Melin et al. (2014) may apply which suggests that tool use and other cognitive skills evolved among frugivorous primates as a strategy to exploit extractable, seasonal fall-back food resources. Tool use for procuring ants, as in chimpanzees, could then emerge as a by-product of a generally higher “sensorimotor intelligence” (Melin et al. 2014) which evolved under the selection pressures outlined by these authors.

In line with Rothman et al. (2014), we conclude that a more detailed examination of patterns of invertebrate predation by primates, including a higher taxonomic resolution of prey items and a better quantification of the role of invertebrates in primate diets and the foraging technics associated with the procurement of invertebrate prey, holds a strong potential for understanding the evolution of dietary strategies in primates.

## Supplementary Information

Below is the link to the electronic supplementary material.

Supplementary file1 (XLSX 39 kb)

Supplementary file2 (DOCX 13 kb)

## Data Availability

The data on which this review is based are provided as Supplementary Information.
