# Supplementary material for: Myrmecovory in Neotropical primates
Source: Primates. 2021 Sep 29;62(6):871–7. doi: 10.1007/s10329-021-00946-2 (PMC8526450; doi:10.1007/s10329-021-00946-2)
Supplement: Supplementary file 2 — Supplementary file2 (DOCX 13 kb) [file 10329_2021_946_MOESM2_ESM.docx]

**Supplementary Table 2. Ant defences**

| **Subfamily** | **Genus** | **Defences** | | |
| --- | --- | --- | --- | --- |
|  |  | **Sting** | **Venoms** | **Other defences** |
| Dolichoderinae | *Azteca* | vestigial to absent, non-functional |  | aggressive; biting; distastefulness; spraying |
|  | *Dolichoderus* | vestigial to absent, non-functional |  | biting; distastefulness; heavy sclerotized exoskeleton with spines |
| Dorylinae | *Eciton* | strongly developed and functional |  | very aggressive biting; major worker caste with hooked mandibles |
|  | *Labidus* | strongly developed and functional |  | very aggressive biting; major worker caste with enormous masticatory muscles |
| Ectatomminae | *Ectatomma* | usually large and strongly developed | strong, painful |  |
| Formicinae | *Camponotus* | absent, replaced by formic acid-projecting system with acidoporus |  | spraying repellent chemical (formic acid); aggressive biting, especially by major worker caste |
| Myrmicinae | *Cephalotes* | reduced, non-functional as weapon |  | thick, hard integument with spines and crests on different body parts; biting; distastefulness; mimetic or warning colours |
|  | *Crematogaster* | sting spatulate, non-functional as weapon |  | distribute repellent chemical |
|  | *Atta* | reduced, non-functional as weapon |  | spines on head and mesosoma; especially major soldiers with huge heads and strong cutting mandibles |
|  | *Daceton* | well developed and functional |  | spines; trap mandibles |
|  | *Pheidole* | well developed and functional |  | major worker caste with strong shearing or crushing mandibles |
| Ponerinae | *Pachycondyla* | usually large and strongly developed | strong, painful |  |
| Pseudomyrmicinae | *Pseudomyrmex* | usually large and strongly developed | strong, painful |  |

**References for Supplementary Table 2**

Andrade ML de, Baroni Urbani C (1999) Diversity and adaptation in the ant genus *Cephalotes*, past and present. Stuttgarter Beitr Naturk, Ser B, 271:1- 889 (defense mechanisms: 827-850)

Bolton B (1994) Identification guide to the ant genera of the world. Cambridge (Mass), Harvard University Press

Hölldobler B, Wilson EO (1990) The ants. Springer, Berlin

Hunt JH (1983) Foraging and morphology in ants: the role of vertebrate predators as agents of natural selection. In: Jaisson P (ed) Social insects in the tropics, vol 2 (Proceedings of the international symposium. International Union for the Study of Social Insects and the Sociedad Mexicana de Entomología). Université Paris-Nord, Paris, pp 83-104

Kugler C (1978) A comparative study of the Myrmicine sting apparatus (Hymenoptera, Formicidae). Studia Entomol 20:413-548

Wheeler WM (1910) Ants – their structure, development and behavior. Columbia University Press, New York
